# Supplementary material for: My experiences with kidney care: A qualitative study of adults in the Northern Territory of Australia living with chronic kidney disease, dialysis and transplantation
Source: PLoS One. 2019 Dec 19;14(12):e0225722. doi: 10.1371/journal.pone.0225722 (PMC6922340; doi:10.1371/journal.pone.0225722)
Supplement: Attachment — Submitted filename: PONE-S-19-23404_Response to Reviewers.doc [file pone.0225722.s001.doc]

Re: PONE-S-19-23404 “My experiences with kidney care: a qualitative study of adults in the Northern Territory of Australia living with chronic kidney disease, dialysis and transplantation”

**Response to report provided by Editor**

**1. When submitting your revision, we need you to address these additional requirements. Please ensure that your manuscript meets PLOS ONE's style requirements, including those for file naming. The PLOS ONE style templates can be found at:**
[http://www.journals.plos.org/plosone/s/file?id=wjVg/PLOSOne_formatting_sample_main_body.pdf](https://aus01.safelinks.protection.outlook.com/?url=http%3A%2F%2Fwww.journals.plos.org%2Fplosone%2Fs%2Ffile%3Fid%3DwjVg%2FPLOSOne_formatting_sample_main_body.pdf&data=02|01|Alan.Cass@menzies.edu.au|21171fb32c6d4191bdbd08d73af3cad5|9f2487678e1a42f3836fc092ab95ff70|0|1|637042691067887839&sdata=83ukJyMvTM48%2F8dHcVBWcBOvF42KP9TwVTX1Hj9Y5pA%3D&reserved=0) and [http://www.journals.plos.org/plosone/s/file?id=ba62/PLOSOne_formatting_sample_title_authors_affiliations.pdf](https://aus01.safelinks.protection.outlook.com/?url=http%3A%2F%2Fwww.journals.plos.org%2Fplosone%2Fs%2Ffile%3Fid%3Dba62%2FPLOSOne_formatting_sample_title_authors_affiliations.pdf&data=02|01|Alan.Cass@menzies.edu.au|21171fb32c6d4191bdbd08d73af3cad5|9f2487678e1a42f3836fc092ab95ff70|0|1|637042691067887839&sdata=sdCz0LZhF9FE5RDUk6Z2K8DOVb1dbbCtBQozRjD8lKE%3D&reserved=0)

Thankyou for this guidance. Appropriate formatting, to meet the requirements of the journal have now been made.

**2. Please provide additional details regarding participant consent. In the ethics statement in the Methods and online submission information, please ensure that you have specified whether consent was written or verbal/oral. If consent was verbal/oral, please specify: 1) whether the ethics committee approved the verbal/oral consent procedure, 2) why written consent could not be obtained, and 3) how verbal/oral consent was recorded.**

Apologies for this oversight. Clarifying information has now been included, on page 6 (from line 112):

*All participants were aged over 18 years old, had the study explained to them, an interpreter offered, and provided informed written consent.*

**3. Thank you for stating the following in the Acknowledgments Section of your manuscript:**

**JH was supported by an Australia National Health and Medical Research Council Fellowship (1092576).**

**We note that you have provided funding information that is not currently declared in your Funding Statement. However, funding information should not appear in the Acknowledgments section or other areas of your manuscript. We will only publish funding information present in the Funding Statement section of the online submission form.**

**Please remove any funding-related text from the manuscript and let us know how you would like to update your Funding Statement. Currently, your Funding Statement reads as follows:**

**JH was awarded the funding for the study, which was funded by Top End Health Services (**[**https://health.nt.gov.au/health-governance/top-end-health-service**](https://aus01.safelinks.protection.outlook.com/?url=https%3A%2F%2Fhealth.nt.gov.au%2Fhealth-governance%2Ftop-end-health-service&data=02|01|Alan.Cass@menzies.edu.au|21171fb32c6d4191bdbd08d73af3cad5|9f2487678e1a42f3836fc092ab95ff70|0|1|637042691067897830&sdata=rY9t3mCUujnZbCf23gvo77dp5BifnK6r3fjzlosPZAI%3D&reserved=0)**)
The funder commissioned the work, but had had no role in study design, data collection and analysis, decision to publish, or preparation of the manuscript.**

We have now removed any reference to funding in the manuscript (page 23, lines 468):

*The interpretation and reporting of these data are the responsibility of the authors and was not influenced by the funder, Top End Health Service. JH was supported by an Australia National Health and Medical Research Council Fellowship (1092576).*

Please update the Funding Statement as follows:

*The study was funded by Top End Health Services (*[*https://health.nt.gov.au/health-governance/top-end-health-service*](https://health.nt.gov.au/health-governance/top-end-health-service)*), who commissioned the work, but had had no role in study design, data collection and analysis, decision to publish, or preparation of the manuscript. JH was supported by an Australian National Health and Medical Research Fellowship (1092576).*

**4.  Thank you for stating the following in the Competing Interests section:**

**This detail is included in the manuscript:**

**I have read the journal's policy and the authors of this manuscript have the following competing interests: grant funding of the study from Top End Health Services for the submitted work; Hughes, Beaton, Wood, Signal, Majoni and Maple-Brown provide clinical care and/ or are department leaders within the study setting (Top-End Renal Service).  There are no other relationships or activities that could appear to have influenced the submitted work.**

**Please confirm that this does not alter your adherence to all PLOS ONE policies on sharing data and materials, by including the following statement: "This does not alter our adherence to PLOS ONE policies on sharing data and materials.” (as detailed online in our guide for authors** [**http://journals.plos.org/plosone/s/competing-interests**](https://aus01.safelinks.protection.outlook.com/?url=http%3A%2F%2Fjournals.plos.org%2Fplosone%2Fs%2Fcompeting-interests&data=02|01|Alan.Cass@menzies.edu.au|21171fb32c6d4191bdbd08d73af3cad5|9f2487678e1a42f3836fc092ab95ff70|0|1|637042691067897830&sdata=DB4oCCmWQCELMgk%2BNG7xmGqeADSw%2BR%2Fsz6lF6EN%2F5N4%3D&reserved=0)**).  If there are restrictions on sharing of data and/or materials, please state these. Please note that we cannot proceed with consideration of your article until this information has been declared.**

**Please include your updated Competing Interests statement in your cover letter; we will change the online submission form on your behalf.**

*Thank you for the opportunity to clarify this information. As included in the cover letter, please update the Competing Interests statement to include:*

*Any enquiries about the research can be directed to the corresponding author on behalf of the research reference group. However, we cannot provide data in a data sharing repository due to following reasons*

1. *The intent of the research was to support increased understanding of the experiences of health care users within this local health service, and there is a high potential for re-identification of individuals given the nature of the research and small number of study participants.*
2. *Participants did not provide consent for their research data to be archived in a data sharing repository*
3. *We respect and support the important principle of First Nations Data Sovereignty, that First Nations peoples’ have rights to control who has access to, and who will share and interpret Indigenous data*

**Please know it is PLOS ONE policy for corresponding authors to declare, on behalf of all authors, all potential competing interests for the purposes of transparency. PLOS defines a competing interest as anything that interferes with, or could reasonably be perceived as interfering with, the full and objective presentation, peer review, editorial decision-making, or publication of research or non-research articles submitted to one of the journals. Competing interests can be financial or non-financial, professional, or personal. Competing interests can arise in relationship to an organization or another person. Please follow this link to our website for more details on competing interests:** [**http://journals.plos.org/plosone/s/competing-interests**](https://aus01.safelinks.protection.outlook.com/?url=http%3A%2F%2Fjournals.plos.org%2Fplosone%2Fs%2Fcompeting-interests&data=02|01|Alan.Cass@menzies.edu.au|21171fb32c6d4191bdbd08d73af3cad5|9f2487678e1a42f3836fc092ab95ff70|0|1|637042691067907827&sdata=qKOMRRzONiasYNLTvabjFEywfaHb1sAfx4eslzRkeZQ%3D&reserved=0)

**5. Please amend the manuscript submission data (via Edit Submission) to include author Cass A.**

Professor Cass has now been included in the manuscript submission data. We have also removed the following sentence in the acknowledgement section at page 23, which starts at line 468:

*“We acknowledge Prof Alan Cass who gave advice on study design, read and participated in review of the manuscript.”*

**Reviewers' comments:**

**Reviewer #1: Hughes et al report their qualitative work on adults with chronic kidney disease based in Darwin, Australia. The work presented describes their methods and their findings, all of which meet the COREQ checklist.** **I have a few minor comments:
- The title suggests northern Australia, yet the work was limited to the top-end of the Northern Territory. Do the authors assume that Torres Strait Islanders would report the same or would a more appropriate title reflect precisely the participant population involved?**

This is a good point. We have changed the title to reflect the Northern Territory context:

*My Experiences with Kidney Care: A qualitative study of adults in the Northern Territory living with chronic kidney disease, dialysis and transplantation*

**- The abstract aim states "To describe the experiences of Aboriginal health care users ...", yet only 81% self-identified as Aboriginal and this specificity was dropped in the final paragraph of the introduction. Could the authors please clarify the aim?**

Thank you for this comment. We have adjusted the aim to accurately reflect the participant group. Page 2, line 27:

*Aim: To describe the experiences of health care users of a large government kidney healthcare service provider.*

**- Selection of participants - was the initial approach made in the participant's language (given 86% of Aboriginal participants had English as second or later language and the researchers were not known to participants).**

While English was not the first language for many participants involved in the study, participants accessing care the renal services involved in the study were fluent in English. Page 6, lines 112-113.

*All participants were aged over 18 years old, spoke English, had the study explained to them in English, an interpreter offered, and provided informed written consent.*

**- How does this work relate to similar findings including patient experience measures? For example the discussion around sharing of knowledge and its relationship to Breckenridge's framework for shared-decision making (Nephrol Dial Transplant 2015, 30: 1605)?**

*Thank you for the opportunity to reflect on the important article by Breckenridge, which talks to efforts to promote the collection and reporting of PROMs and PREMs to improve understanding of renal patients’ experiences. Reporting a survey of activity amongst European renal registries, findings of a systematic review, and views of experts presented at a consensus forum of health professionals, it suggests certain PROMs instruments might be appropriate for routine renal registry collection (SF-12, the disease-specific KDQOL-36 and EQ-5D). We note the this consensus meeting failed to acknowledge concerns that these QoL instruments do not include dimensions of quality of life that are likely to be relevant to Indigenous Australians .*

*This research project was developed as there was no data tool to record and describe the health care experience within this population, which has a high proportion of Indigenous Australian clients. We recognise the importance of shared decision making (centred on the client health and wellbeing) which requires an exchange of information, preferences and values about treatments, discussion and agreement about a treatment plan. We also recognise that such understanding will not be provided through the use of conventional QoL tools, but with careful qualitative research to understand Aboriginal patients’ views about their treatment such as that undertaken in this study, and supported by findings outlined by others . This type of research is required first, and may later be co-designed and developed into categorical survey instruments, to enhance other health outcomes data repositories.*

*We have slightly re-ordered the original text to accommodate this recommended inclusion, and included the following text into the existing paragraph in the discussion section starting at line 413:*

*We note reporting a survey of activity amongst European renal registries, findings of a systematic review, and views of experts presented at a consensus forum of health professionals, suggest certain patient-related outcomes measures (PROM) instruments might be appropriate for routine renal registry collection (SF-12, the disease-specific KDQOL-36 and EQ-5D) . It is also recognised that existing PROM instruments lack validity within Indigenous populations, between non-Indigenous and Indigenous populations, and lack consideration of the holistic and collective health frameworks of Indigenous Australian peoples operating within western models of health care .*

*The following is added at line 422:*

*We also note resources are needed (such as shared consultation spaces) for health care providers and users exchange knowledge and enhance patient-level health care decision making . A shared space which allowed recognition and synthesis of these priorities was built within our study governance structure , and enabled identification of priorities which sitting within and outside of the remit of the health service (health care provider professionalism versus urban and remote accommodation and housing policy). Currently, our national renal registry, ANZDATA, examine patient-level health outcomes and lack mechanisms to record and signpost health system policy shifts, individual health service capability, resourcing and workplace culture which may explain patient patient-level health outcomes or experiences. This may be an important Registry innovation.*

**Reviewer #2: This manuscript describes the experiences of Aboriginal health care users of the Top End Health Services' renal services in Northern Territory. Although it seemed to be a service improvement exercise, it is important research and findings will inform policy changes. I nonetheless have a few suggestions for the researchers' considerations;**

**1. I encourage the researchers to use qualitative reporting checklists like the Standards for Reporting Qualitative Research (SRQR) or the COnsolidated criteria for REporting Qualitative research (COREQ) to guide their writing.**

Thank you for this suggestion. We did use the COREQ checklist in the finalisation of this manuscript and can provide this to the Editor if required.

**2. A detailed description of the Australian Indigenous community's health and services gaps and, in particular, the situation in the Top End are necessary for context for an international audience.**

To improve the flow of the manuscript, two paragraphs with guiding headings have been included after the first paragraph of the introduction (starting at page 4, lines 61-84). We have also slightly altered the adjacent sections to improve communication of information after inserting the following text:

***Chronic and end stage kidney disease***

*Chronic kidney disease (CKD) is a term describing sustained impairment of kidney function, and indicated biochemically by low glomerular filtration rate and/ or albuminuria, and early disease is often asymptomatic. Advanced or end stage kidney disease (ESKD), when there is very impaired kidney function, is associated with increasing symptom and requires dialysis and/ or kidney transplantation to sustain life. ESKD treatment is publicly funded in Australia and is provided to more than 25,000 Australians at a cost of more than $1 billion per year.*

***Indigenous Australians living in regional and remote settings have high ESKD care needs***

*Nationally, Aboriginal and Torres Strait Islander peoples (Indigenous Australians) use renal replacement therapy (RRT) to manage ESKD at a 5 times higher rate than other Australians , and have lower access to home-based renal replacement therapy including kidney transplantation . The Northern Territory (NT) of Australia is reported to have the highest national prevalence of dialysis dependent ESKD of 2812 v 531 per million population compared to other Australians , and the highest incidence of dialysis requiring ESKD among First Nations peoples living across Australian states and Territories at 1344 per million population . In the NT, adults with kidney disease have heavy hospital care needs , and recent modelling has projected a linear increase in ESKD in this region over the coming decade , which is further supported by evidence of rapid annual decline in kidney function in Aboriginal and Torres Strait Islander adults . However, many Territorians who have the highest need of ESKD renal care services, live in remote and very remote regions, and are required to relocate over vast distances to access centrally located nurse-assisted-haemodialysis models of care health care services . Advocacy by clients, families, and renal health care clinicians have highlighted this progressive burden of CKD among Indigenous Australians , which has been recently supported by nationally targeted policy and funding responses .*

**3. The use of a phenomenological approach, face-to-face interviews and focus groups in this case were appropriate but it would still be useful to know the pros and cons of using this as opposed to other methodologies in an Indigenous population and, in particular again, in the Top End.**

As recognised, phenomenology was an appropriate research methodology for this study as it enabled participants’ experiences of Kidney care in the Top End to be explored (i.e. the essence of experience). Other qualitative methodologies would also have been suitable (for example grounded theory). These approaches can be coupled with methods that are suitable for working with Aboriginal and Torres Strait Islander people. In this instance, some of the strategies employed to ensure this methodology was respectful to working with this population, in the Top End context, included: guidance from the Indigenous Reference Group, use of open-ended interview questions, iterative data analysis, and open-coding involving multiple people.

**4. There should be a description of the credentials, occupations, experience and training of each researcher and their relationships with the participants to give a sense of possible biases and assumptions.**

Apologies for this oversight. Please find the additional information on page 6 (lines 115-124):

*The investigator team for this study included patients, community members, health care providers and researchers with clinical, health services and social science expertise. Of the team, JH is an Indigenous nephrologist and research fellow who co-lead the project in collaboration with RK a non-Indigenous social scientist (PhD in Public Health). BB is a First Nations person from Papua New Guinea and experienced renal services senior Indigenous Liaison Officer. Co-authors, AMP, MH and GT, were members of the Indigenous Reference Group, all of whom speak Aboriginal languages, and have lived experiences of renal replacement care services. NF is a non-Indigenous researcher with expertise in qualitative research (Masters of Evaluation) who was instrumental in data collection and analysis.*

**5. There was no mention of software used - coding and analysis were all managed manually?**

Please find the following information to clarify details around data analysis (page 8, lines 155-156):

*Transcripts were coded by three members of the research team (NF, BB, RK). NF and RK used NVivo (version 11) to manage data analysis, while BB coded transcripts manually. Data was managed in a secure Excel file.*

**6. It is not clear exactly HOW the analysis occurred. I read reference no. 11 and still did not fully understand whether the FGs were conducted to co-interpret the interview data OR the FGs were also part of the data that was analysed. I suggest rewriting that paragraph in Methods.**

Apologies for the lack of clarity around this aspect of the study. Additional information has now been included and the existing paragraph has been separated into two in an effort to make this clearer (page 8, lines 146-147):

*The synthesis and co-interpretation of patient experience was developed in four focus group meetings with Indigenous dialysis clients, in a non-clinical urban setting between April 2017 and November 2017. The development of the focus groups has been described elsewhere . Participants in these recurring focus group meetings provided written consent for each focus group to be audio recorded and externally transcribed to further inform findings from this study. These focus group discussions were guided by content raised in previous focus groups and emerging findings identified from anonymised interviews.*

*Transcripts from interviews and focus groups were returned to participants who requested this at the time of consent. Transcripts were coded by three members of the research team (NF, BB, RK).* *NF and RK used Nvivo (version 11) to manage data analysis, while BB coded transcripts manually.*

**7. Also, the researchers said that the second coding round was based on existing theories but did not say WHICH theories and it is not apparent in Results or Discussion that any theory was used.
Data saturation was not achieved in all themes - there was no description of how this was addressed.**

Apologies for the lack of clarity around coding. Please find the following information (page 9, lines 157-163):

*The first round of coding was inductive, to produce new understandings of patient experiences and satisfaction of renal care. The second coding round was deductive based on constructs as directly related to the interview guide, such as satisfaction with renal services (including transport, care received, relationships with staff). The final focus group meeting enabled the accuracy of data interpretation to be cross-checked with participating members. While saturation was reached on many constructs, many contradictory cases were identified which contributed valuable information to this study.*

**8. Please do not use 'n=...' to describe the proportion of respondents in a qualitative paper.**

*This was used in the section related to types of kidney related conditions of client participants and has now been removed (i.e. page 9, line 176):*

*The kidney related conditions of client participants included CKD (11, 42.3%)*

**9. Some of the quotes are quite intriguing but they are not identified (eg. by participant no.) and some of the themes are poorly supported by quotes such as the 'Care and supporting environments' theme.**

*We have included identifiers in this format [xxx] at the end of quotes*

**10. Some of the themes are not consistent with the findings or quotes presented. Eg. Communication and Information - this seems to be more about community understanding of their conditions which may or may not be compounded by communication with and information from health care providers; Commitment and Care Proficiency - this does not describe commitment at all but rather competence of staff and continuity of care; Care and supporting environments - this seems to be describing standards of support and confidentiality. The logic of grouping of these themes are also not apparent to me. I actually thought that the 4 factors described in 1st paragraph in Discussion (knowledge gaps, relocation impact, health care providers' quality, service environments) would serve much better and clearer as themes. I also suggest the use of subthemes to further signpost in the Discussion section.**

Thank you, we agree that the four factors described in the first paragraph of the discussion are a better way to express the experiences of respondents. As such we have amended the main results themes as follows:

1) Knowledge gaps, 2) Impacts of relocation, 3) Service environment, 4) Health Care Provider quality. Guiding headings have been included in the manuscript in the introduction, and headings indicating sub-themes have now also been incorporated throughout the discussion.

**11. Discussion was fairly well-written. But, again, the use of theories, if any, to interpret and synthesize the findings should be discussed.**

*Thank you again for raising this point. As has now been made clearer in the methods section, constructs guiding the interviews were deductively analysed for in the second round of coding. Existing literature has been discussed in relation to these findings in the discussion.*

**12. I think the first few lines in Conclusion should summarize the answer to the aim of this research - what are the experiences of renal patients using TEHS' renal services.**

We have now included the following sentence in the conclusion:

*Four major themes identified by clients related to their experience with renal care provided by this major regional health care provider: knowledge gaps of their own condition, the lived impacts of relocating to access health care, service environments, and Health Care Provider Quality.*

**13. The last sentence in Conclusion should really be under strengths and limitations. It isn't a conclusion!**

*We agree and have moved this sentence to the end of the strengths section (page 23, lines 461-464).  We have also ensured the conclusion is now consistent across the abstract and main manuscript.*
